# Do illness beliefs predict uptake of depression treatment after web-based depression screening? A secondary analysis of the DISCOVER RCT

**DOI:** 10.1136/bmjment-2025-301666

**Published:** 2025-07-27

**Authors:** Matthias Klee, Franziska Sikorski, Bernd Loewe, Sebastian Kohlmann

**Affiliations:** 1 Department of General Internal Medicine and Psychosomatics, Heidelberg University Hospital, Heidelberg, Germany; 2 Department of Psychosomatic Medicine and Psychotherapy, University Medical Center Hamburg-Eppendorf, Hamburg, Germany

**Keywords:** Depression, Depression & mood disorders

## Abstract

**Background:**

Only a minority of those with depressive disorder receive treatment. Besides system-level factors, individual factors could account for the gap between detection and treatment of depression in so far unreached but affected populations.

**Objective:**

This study tests the predictive value of illness beliefs (IB) for the uptake of depression treatment 6 months after web-based depression screening.

**Methods:**

This is a secondary analysis of the randomised controlled Germany-wide DISCOVER trial that investigated the effects of automated results feedback following web-based depression screening in untreated participants with at least moderate depression severity (Patient Health Questionnaire ≥10 points). IB were examined as predictors of depression treatment uptake. Eligible participants were at least 18 years old, reported proficiency in German language, and provided informed consent. IB were assessed at the time of screening (baseline) with an adapted version of the Brief Illness Perception Questionnaire. Uptake of depression treatment was operationalised as self-reported initialisation of psychotherapy and/or antidepressant medication 6 months after baseline. Analyses were adjusted for study arm.

**Findings:**

Data from N=871 participants of the DISCOVER trial providing follow-up data were analysed. IB denoting more consequences (OR (95% CI) 1.12 (1.00 to 1.26)), higher treatment control (OR (95% CI) 1.19 (1.11 to 1.29)) and a depression-conforming illness identity (OR (95% CI) 1.65 (1.15 to 2.36)) were associated with up to 56.8% relative increase in predicted probability of depression treatment uptake 6 months after baseline.

**Conclusions:**

Results suggest considerable effects of IB for depression treatment uptake in untreated populations.

**Clinical implications:**

IB could reflect relevant barriers in access to depression care and, concurrently, intervention targets to foster health service utilisation in untreated populations.

**WHAT IS ALREADY KNOWN ON THIS TOPIC**

- Illness beliefs (IB) have been identified as determinants of health behaviour in clinical populations. However, little is known about the association between IB and health behaviour in untreated populations with moderate depression severity.

**WHAT THIS STUDY ADDS**

- This study demonstrates that IB predict uptake of depression treatment 6 months after web-based depression screening in a currently untreated online population of people with at least moderate depression severity.

**HOW THIS STUDY MIGHT AFFECT RESEARCH, PRACTICE OR POLICY**

- IB are significant individual-level determinants of depression treatment uptake, highlighting potential barriers to accessing care. Future research could explore these barriers and develop strategies to improve treatment access for people with depression who remain untreated.

## Background

 The widening gap between the number of individuals with prevalent depression and the number of individuals receiving care challenges the population-level impact of advancements in recognition and treatment of depression.^1^ Depression screening of the adult population or screening of individuals with risk factors has been recommended by guidelines but also critically discussed within the research community.^2 3^ Zooming in on potential consequences, evidence from the USA and Germany indicates that approximately only every third patient affected by depression receives or initiates depression treatment in settings with systematic depression screening.^4^,^6^ As such, the question of what factors drive the uptake of depression treatment appears pressing.

Besides system-level factors such as recognition, referral or availability of mental healthcare, individual-level factors were previously considered as determinants of help seeking in people with depression.^7^ Reported factors capture barriers in access to care (younger age, male sex, ethnic minority status) or need (higher symptom severity, longer episode duration) but characterise risk groups rather than capturing underlying mechanisms.^7^ Potential mechanisms may be captured with cognitive representations of experienced symptoms underlying illnesses. Illness beliefs (IB) were associated with adherence to treatment in adults undergoing haemodialysis and with clinical outcomes such as coronary heart disease severity or glycaemic control.^8^,^11^ Conceptually, IBs reflect an individual’s understanding of an illness. IB can foster beneficial health behaviour and are inherently related with patient journeys and prognostic health outcomes such as emotional distress, recovery or survival.^12^ Knowledge on the role of depression-related IB for behavioural outcomes is scarce, despite some evidence for their role for self-management and mental health outcomes.^13^

Compared with somatic diseases, symptoms of depression are heterogeneous in nature and less specific. This representation could affect a person’s ability to recognise and attribute symptoms to a depressive disorder, resulting in a lower likelihood to seek help.^14^ Contrary, if symptoms are recognised and correctly attributed to an underlying depression, and if depression is cognitively represented as a condition that can be effectively treated by a healthcare professional, help seeking may be fostered, irrespective of system-level barriers in access to care. Consequently, health service research may benefit from a deeper understanding of how IB relate to help-seeking behaviour and treatment initiation, with IB reflecting a bottleneck preceding recognition of or screening for depression and consequent treatment in primary care.^7 15 16^

So far, research focusing on IB has focused on primary care settings and clinical outcomes, with behavioural consequences mainly relating to engagement in or adherence to treatment.^9 13^ However, models of help-seeking such as the Common Sense Model of Self-Regulation allude to the role of sociocultural context for IB formation and hence denote antecedents of IB manifesting even before (self-)identification or recognition in primary care.^17 18^ Consequently, IB in people living with depression that did not reach primary care yet are not captured by previous research, despite their importance for fostering access to care.

Moving one step before help-seeking in primary care, most people seek mental health information online, especially in areas with lower availability, that is, higher system-level barriers.^19 20^ Targeting individuals that seek information online is an important use case to further elucidate the role of IB for treatment uptake in a high-risk population that did not yet reach primary care.

### Objective

The aim of this study was to investigate the role of IB for the uptake of depression treatment 6 months after web-based depression screening in untreated individuals with at least moderate depression severity.

## Methods

### Study sample and design

In this secondary analysis of the randomised controlled trial (RCT) DISCOVER,^4^ IB were examined as prospective predictors of depression treatment uptake 6 months after web-based depression screening.

The primary aim of the observer-masked, three-armed DISCOVER RCT in Germany was to investigate the effect of automated results feedback following web-based depression screening in individuals with at least moderate depression severity (Patient Health Questionnaire-9 (PHQ-9) ≥ 10), who had not received a diagnosis of depression or treatment for depression in the preceding year. Eligible participants were at least 18 years old, reported proficiency in German language, and provided online informed consent via checkboxes on the study website.^4^ Previously published findings of the DISCOVER RCT showed that automated feedback following web-based depression screening neither reduces depression severity nor leads to sufficient depression care.^4^

By design, participants treated for depression currently or within the preceding year were excluded prior to randomisation. Participants were randomised to either receive non-tailored, tailored or no feedback (1:1:1) following web-based depression screening (referred to as baseline hereafter) and were followed-up 6 months after baseline.^4^ Baseline assessments were conducted between 12 January 2021 and 31 January 2022. For a more detailed overview of trial procedures and feedback interventions, see Kohlmann *et al*^4^ or Sikorski *et al*.^20^ The study followed appropriate Consolidated Standards of Reporting Trials guidelines.^20^

Baseline assessments including the depression screening were conducted via web-based self-report questionnaires. Two to 5 days after baseline assessment, participants were contacted via telephone for complementary diagnostic interviews. Six months after baseline, web-based follow-up assessments were set.

### Main outcomes and measures

#### Uptake of depression treatment

The primary outcome uptake of depression treatment was operationalised as self-reported initiation of psychotherapy and/or pharmacotherapy with antidepressant medication until 6 months after baseline.^4^ Uptake of depression treatment was assessed with two questions ‘Have you started any psychotherapy or similar treatment in the last 6 months?’ and ‘Have you started taking medication to treat depression or other complaints such as sleep problems, anxiety or stress?’.

#### Secondary outcomes

Secondary outcomes comprised indicators of health behaviour and symptom severity change, assessed 6 months after baseline. Behavioural outcomes were self-reported receipt of a diagnosis of major depression as well as engagement in depression-related health behaviour (DRHB), with categories social (seeking help with family, friends or self-help groups), manage (reading self-help books, engaging in physical activity or relaxation), help (seeking contact to a medical or health professional) or info (seeking information, engaging in online forums). Symptom change was assessed regarding depression severity (PHQ-9), somatic symptom severity (Somatic Symptom Scale-8), anxiety severity (Generalized Anxiety Disorder Scale-7) and health-related quality of life (EQ-5D-5L visual analogue scale).

#### Illness beliefs

IB were assessed at baseline with an adapted version of the B-IPQ.^21^ B-IPQ assessment followed depression screening with the PHQ-9 in order to capture the IB about depressive symptoms.

The B-IPQ is a 9-item instrument assessing five cognitive domains (consequences, timeline, personal control, treatment control and illness identity), two emotional representations (emotions and concern), illness comprehensibility, and causal representations (open self-reported causal attributions) constituting IB (online supplemental table S1). Items measure agreement with statements referring to the individual domains on a scale from 0 to 10, except for the open question about causal representations.^21^ Previous work suggested acceptable psychometric properties of the B-IPQ in the research setting.^9^

Assessment of IB in individuals with at least moderate depression severity without a diagnosis of depression disorder required adapting the operationalisation of illness identity (adapted from: ‘How much do you experience symptoms from your illness?’ to ‘Can you imagine that you are currently suffering from depression?’) and the inclusion of an item operationalising temporality, that is, ‘Is this the first time in your life that you are experiencing such complaints?’. We argue that illness identity is still captured by the adapted item as it assesses the subjective appraisal of symptoms belonging to a specified illness (ie, depression). The remaining items were translated into German, additionally adapting the term of ‘your illness’ to ‘these complaints’, again due to eligible participants not yet having received a diagnosis at baseline (online supplemental table S1).

#### Covariates

Covariates comprised depression severity (PHQ-9), single-item questions regarding previous diagnosis with burnout or depression disorder and depression treatment preference (general practitioner/psychotherapist or unspecified) as well as further demographic characteristics such as age, gender (male or female), formal education (low (<10 years), middle (≥10 years) or high (university entrance qualification)), working status (not working, retired, unemployed, shift work/hourly rate or employed) and living status (with others or alone) assessed at baseline. Covariates further included the presence of a major depressive disorder 2–5 days after baseline, assessed using the Structured Clinical Interview for DSM-5 Disorders. The SCID was conducted via telephone by MSc psychology students trained and supervised by a senior psychotherapist (PhD).

### Statistical analysis

The primary analysis consisted of two logistic regression models, which were specified to examine prognostic validity of IB items at baseline for uptake of depression treatment 6 months after web-based depression screening. Both models incorporated baseline characteristics and the SCID result as covariates and uptake of depression treatment assessed 6 months after web-based depression screening as outcome. The base model comprised baseline characteristics and the SCID result only. The main model included baseline characteristics, the SCID result and IB items. To adjust for design effects of the RCT, both models were adjusted for study arms. Model coefficients were estimated in the primary analysis set, with data from participants without missing information on covariates or the primary outcome.

ORs and 95% CIs were computed. Variance explained was estimated with McFadden’s pseudo-*R^2^*.^22^ Furthermore, model performance was assessed with the area under the receiver operator characteristic curve (AUC).

Sensitivity analyses comprised repeated analysis without SCID interview results as covariate, since 137 of 146 participants removed from the primary analysis set due to missing data were missing SCID interview. Variance-inflation factors were examined in the primary analysis model, to assess multicollinearity. Post hoc power analysis was conducted with G*Power V.3.1.9.7 based on available sample size, type 1 error probabilities, observed ORs, marginal outcome probabilities, IB variance explained and distributional characteristics of the predictor. In exploratory analyses, the validity of findings was assessed with regard to secondary outcomes. The exploratory analysis sets included participants from the primary analysis set with complete data on the respective outcome. In case of analyses examining symptom severity changes, models were additionally adjusted for baseline measures of the respective index. Additionally, exploratory analyses were conducted to assess variations in responses to IB across baseline characteristics using a correlation matrix. For this analysis, categorical variables were dummy-coded to enable interpretation of correlation coefficients.

All analyses were conducted in R V.4.3.1. The secondary data analysis was preregistered and is publicly available at https://doi.org/10.17605/OSF.IO/DTPVE.

## Findings

### Descriptive characteristics

Of 1017 participants included in the primary analysis of the DISCOVER RCT with a positive depression screening, n=871 (85.6%) were included in the primary analysis set. Exclusions were due to missing values regarding uptake of depression treatment (n=1), IB (n=1), working status (n=1) or SCID interview (n=137). Furthermore, data from participants not reporting male or female gender were removed prior to analysis due to small cell size (n=6). Participants were 37.5 (SD=14.0) years old, with n=634 (72.8%) participants reporting female gender.

After 6 months, uptake of depression treatment was reported by 233 (26.8%) participants (table 1). IB regarding consequences, timeline, personal and treatment control, concern and emotional response differed between groups, with higher expressions for all IB but personal control in participants that reported depression treatment uptake. Individuals who initialised treatment reported more often that their symptoms may be due to depression (depression-conforming illness identity IB); the uptake of depression treatment was considerably higher among participants with depression-conforming illness identity IB, at 35.8%, compared with 20.4%, among those without depression-conforming illness identity IB.

**Table 1:** Descriptive characteristics

Characteristic	No depression treatment at follow-up number (%) = 638 (73.2)	Depression treatment at follow-up number (%) = 233 (26.8)	P value
Age, mean (SD)	36.4 (13.9)	40.4 (13.8)	<0.001
Gender			0.99
Male, number (%)	173 (27.1)	64 (27.5)	
Female, number (%)	465 (72.9)	169 (72.5)	
Formal education			0.78
Low (<10 years), number (%)	103 (16.1)	41 (17.6)	
Middle (≥10 years), number (%)	190 (29.8)	72 (30.9)	
High (university entrance qualification), number (%)	345 (54.1)	120 (51.5)	
Working status			0.002
Not working, number (%)	93 (14.6)	23 (9.9)	
Retired, number (%)	44 (6.9)	25 (10.7)	
Unemployed, number (%)	24 (3.8)	20 (8.6)	
Shift work/hourly rate, number (%)	195 (30.6)	54 (23.2)	
Employed	282 (44.2%)	111 (47.6%)	
Living status			0.63
With others, number (%)	432 (67.7)	153 (65.7)	
Alone, number (%)	206 (32.3)	80 (34.3)	
Treatment preference			<0.001
Unspecified, number (%)	170 (26.6)	30 (12.9)	
General practitioner or psychotherapist, number (%)	468 (73.4)	203 (87.1)	
Previous lifetime diagnosis of depression/burnout			<0.001
No previous diagnosis, number (%)	398 (62.4)	112 (48.1)	
Previous diagnosis, number (%)	240 (37.6)	121 (51.9)	
Depression severity (PHQ-9), mean (SD)	14.5 (3.83)	15.2 (4.08)	0.03
SCID interview			<0.001
Negative, number (%)	263 (41.2)	64 (27.5)	
Positive, number (%)	375 (58.8)	169 (72.5)	
Condition			0.21
No feedback, number (%)	229 (35.9)	69 (29.6)	
Non-tailored feedback, number (%)	205 (32.1)	80 (34.3)	
Tailored feedback, number (%)	204 (32.0)	84 (36.1)	
Illness beliefs			
Consequences,* mean (SD)	6.20 (2.05)	6.92 (1.94)	<0.001
Timeline,* mean (SD)	6.51 (2.27)	6.94 (2.25)	0.01
Personal control,* mean (SD)	4.99 (2.19)	4.63 (2.18)	0.03
Treatment control,* mean (SD)	5.87 (2.70)	7.14 (2.34)	<0.001
Concern,* mean (SD)	6.31 (2.42)	7.06 (2.16)	<0.001
Comprehensibility,* mean (SD)	6.57 (2.35)	6.59 (2.34)	0.90
Emotions,* mean (SD)	7.23 (2.04)	7.81 (1.80)	<0.001
Temporality (previous experience vs first time),† number (%)	504 (79.0)	183 (78.5)	0.96
Illness identity (yes vs no/maybe),‡ number (%)	231 (36.2)	129 (55.4)	<0.001

* Items are rated from 0 to 10 with 10 reflecting highest agreement with statements listed in Table S1online supplemental table S1.

† Binary item indicating if participants experienced the complaints before.

‡ Binary item indicating if participants appraise their symptoms to probable depression.

PHQ-9, Patient Health Questionnaire-9; SCID, Structured Clinical Interview for DSM Disorders.

Furthermore, participants that initialised depression treatment were older, with distinct working status, more frequently reported a treatment preference for either general practitioners or psychotherapists, more frequently reported a previous diagnosis of depression or burnout, reported higher depression severity and were more likely to fulfil SCID criteria of major depressive disorder.

### IB and uptake of depression treatment

More consequences (How much do these complaints affect your life?; OR (95% CI) 1.12 (1.00 to 1.26)), higher treatment control (How much do you think a treatment can help with these complaints?; OR (95% CI) 1.19 (1.11 to 1.29)) and a depression-conforming illness identity (Can you imagine that you are currently suffering from depression?; OR (95% CI) 1.65 (1.15 to 2.36)) were positively associated with the probability of uptake of depression treatment 6 months after screening (table 2). In line with recommendations by Grant,^23^ we computed the range of percent increase in uptake of depression treatment per 1-point increase of IB in their respective scale across deciles of the predicted baseline probability of uptake of depression treatment. As such, our results suggest a relative increase in predicted probability of depression treatment uptake between 5.6% and 10.9% per 1-point increase of IB regarding consequence, between 8.6% and 17.2% per 1-point increase of IB regarding treatment control and between 24.2% and 56.8% in case of a depression-conforming illness identity IB. Inclusion of IB items in the base model increased McFadden’s pseudo-*R*^2^ from 0.066 to 0.118, suggesting improved model fit in the primary analysis model. Furthermore, discrimination was improved in the primary analysis model (AUC (95% CI) 0.73 (0.69 to 0.76)) compared with the covariate only model (AUC (95% CI) 0.68 (0.64 to 0.72); online supplemental figure S1).

**Table 2:** OR for uptake of depression treatment 6 months after web-based depression screening

Term	OR	SE	P value	Lower CI	Upper CI
Illness belief					
Consequences*	1.12	0.06	0.04	1.00	1.26
Timeline*	0.98	0.05	0.61	0.89	1.07
Personal control*	0.94	0.04	0.11	0.86	1.01
Treatment control*	1.19	0.04	<0.001	1.11	1.29
Concern*	0.98	0.05	0.75	0.89	1.09
Comprehensibility*	0.96	0.04	0.35	0.89	1.04
Emotions*	1.04	0.06	0.48	0.93	1.17
Temporality (previous experience)†	1.05	0.22	0.81	0.69	1.63
Illness identity (yes)‡	1.65	0.18	0.01	1.15	2.36
Condition					
No feedback	Reference				
Non-tailored feedback	1.35	0.21	0.15	0.90	2.03
Tailored feedback	1.40	0.20	0.10	0.94	2.09
Depression severity (PHQ-9)	0.96	0.03	0.16	0.91	1.01
Treatment preference					
Unspecified	Reference				
General practitioner or psychotherapist	2.06	0.23	0.002	1.32	3.31
Previous lifetime diagnosis of depression/burnout					
No previous diagnosis	Reference				
Previous diagnosis	1.32	0.19	0.13	0.92	1.91
Formal education					
Low (<10 years)	Reference				
Middle (≥10 years)	1.07	0.27	0.79	0.64	1.82
High (university entrance qualification)	1.23	0.27	0.44	0.73	2.12
Gender					
Female	Reference				
Male	1.08	0.20	0.70	0.73	1.59
Age	1.02	0.01	0.03	1.00	1.03
Living status					
With others	Reference				
Alone	1.08	0.18	0.69	0.75	1.53
Working status					
Not working	Reference				
Retired	1.45	0.42	0.38	0.63	3.36
Unemployed	2.50	0.42	0.03	1.09	5.74
Shift work/hourly rate	0.89	0.30	0.71	0.50	1.64
Employed	1.23	0.28	0.47	0.71	2.18
SCID screening					
Negative	Reference				
Positive	1.60	0.19	0.01	1.10	2.33

* Items are rated from 0 to 10 with 10 reflecting highest agreement with statements listed in Table S1online supplemental table S1.

† Binary item indicating if participants experienced the complaints before. With the reference level *first time*, OR indicates greater uptake in those with previous experience.

‡ Binary item indicating if participants appraise their symptoms to probable depression. With the reference level *no/maybe*, OR indicates greater uptake in those with a depression-conforming illness identity belief.

GP, general practitioner; PHQ-9, Patient Health Questionnaire-9; PT, psychotherapist; SCID, Structured Clinical Interview for DSM Disorders.

Sensitivity analysis suggests no critical level of multicollinearity with either model specification (all variance-inflation factors <2). Coefficients for covariates were similar in the main and the base model of the primary analysis, except for previous diagnosis of depression or burnout, which was not significantly associated with uptake of depression treatment when including IB (online supplemental table S2). Descriptive statistics for participants lost to follow-up are depicted in online supplemental table S3. Since missingness was largely driven by missing SCID interview results, and since missingness was not related with depression severity, we used complete case analysis and ran further sensitivity analyses. The main model yielded a similar pattern of results when estimated without SCID interview result as covariate, reducing drop-out to n=12 (online supplemental table S4). Differences to IB estimates from the main analysis were a now non-significant but similar point estimate for IB regarding consequence and a similar but now significant point estimate for IB regarding personal control. Post hoc power analyses suggested sufficient power to detect medium effects, but insufficient power to detect small effects for continuous (with OR <1.12) IB (online supplemental table S5).

### IB and secondary outcomes

**Figure 1:**
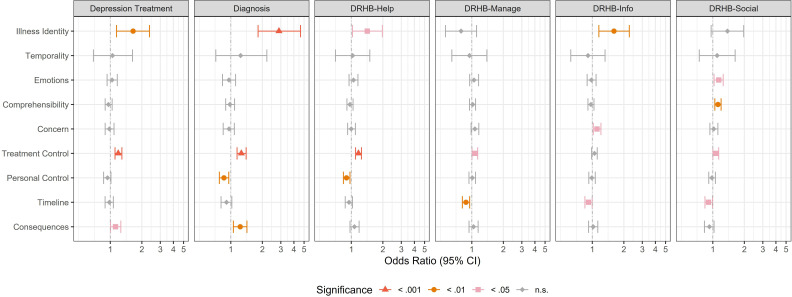
Coefficients for illness beliefs in regression models with primary and secondary dichotomous outcomes. Significance at p<0.05 is denoted with pink colour, significance at p<0.01 is denoted with orange colour, significance at p<0.001 is denoted with red colour. Diagnosis, diagnosis of burnout or depression during follow-up; DRHB, depression-related health behaviour.

Validity of findings was assessed in an exploratory analysis of the role of IB for secondary outcomes. Higher odds of behavioural outcomes (having received a diagnosis of burnout or depression during follow-up, exhibiting DRHB) were identified for higher treatment control (except for DRHB-Info) and consequences (except for DRHB domains) as well as a depression-conforming illness identity IB (except for DRHB-Social and Manage) (figure 1). A longer timeline belief was associated with lower odds of DRHB-Manage/DRHB-Info and DRHB-Social, with a similar although non-significant pattern for uptake of depression treatment, receipt of a diagnosis and DRHB-Help. Temporality was not associated with any secondary outcome (online supplemental tables S6–S14). More intense emotional representation was associated with higher odds of DRHB-Social (emotions) and DRHB-Info (concern). Higher personal control was associated with lower odds of receipt of a diagnosis and DRHB-Help. Higher comprehensibility was associated with higher odds of DRHB-Social.

**Figure 2:**
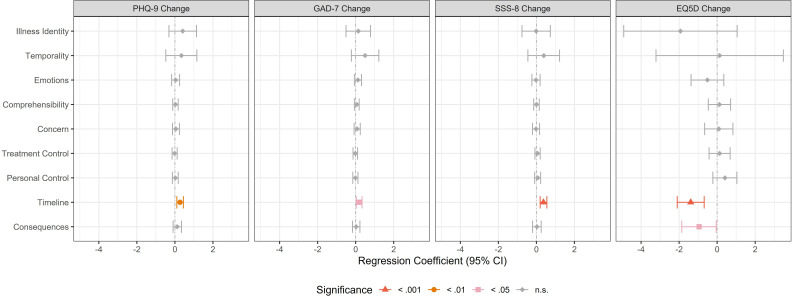
Coefficients for illness beliefs in regression models with continuous secondary outcomes. Significance at p<0.05 is denoted with pink colour, significance at p<0.01 is denoted with orange colour, significance at p<0.001 is denoted with red colour. Note that except for the EQ-5D, higher values reflect less favourable change. EQ-5D, health-related quality of life assessed with the EQ-5D-5L visual analogue scale; GAD-7, anxiety severity assessed with the Generalized Anxiety Disorder Scale-7; PHQ-9, depression severity assessed with the Patient Health Questionnaire-9; SSS-8, somatic symptom severity assessed with the Somatic Symptom Scale-8.

IB were not associated with changes in symptom severity 6 months after web-based depression screening (somatic symptom severity, depression severity, anxiety severity, health-related quality of life), except for timeline (associated with less favourable change on all scales) and consequences (associated with less favourable change in health-related quality of life) (figure 2).

### Response patterns for IB

Examining covariate profiles across IB responses, correlations were low to moderate ranging from r=−0.24 to r=0.29, with correlations |r| ≥0.2 for age, higher education, previous diagnosis of depression or burnout, SCID interview result and being retired (online supplemental figure S2). Higher education was associated with lower IB except for personal and treatment control. Previous diagnosis of depression or burnout was associated with higher IB except for temporality. Positive SCID interview result was associated with higher IB except for personal control and temporality. Higher age and being retired were associated with higher IB except for treatment control and temporality.

## Discussion

### General findings

To our knowledge, this is the first study examining the predictive validity of depression-related IB for the uptake of depression treatment in a currently untreated population with at least moderate depression severity. We found that IB, specifically IB expressing more consequences, higher treatment control and a depression-conforming illness identity, were associated with higher depression treatment uptake 6 months after web-based depression screening. In particular, participants endorsing a depression-conforming illness identity showed up to 56.8% relative increase in the predicted probability of depression treatment uptake compared with those without such beliefs, suggesting that IB may play a substantial role for depression treatment uptake in untreated populations.

### IB and uptake of depression treatment

Our findings are in line with a previous study that investigated uptake of guideline-concordant depression treatment after depression screening in a primary care sample of veterans.^16^ The authors reported that higher external control beliefs and illness identity (ie, reporting that symptoms were unrelated to depression) predicted depression treatment 3 months later. Importantly, endorsed causal beliefs about determinants of experienced symptoms may affect if people appraise their symptoms as depression-conforming (illness identity) or not and determine the health service at which they seek for help.^24^ Previous research found distinct frequencies of causal attributions in people with depression who are enrolled in routine care for a comorbid cardiac disease opposed to those enrolled in routine care for depression.^25^ It is unclear if IB act as determinants of self-selection into, that is, routine care settings or if care settings affect IB formation. Expanding previous findings, our results support IB as determinants for the uptake of depression treatment already prior to contact with clinical entities. This is reflected in the consistency of IB that predict future depression treatment uptake in primary care, and IB that we identified in an online population of currently untreated individuals. Comparing IB patterns that predicted uptake of depression treatment across settings, expressed IB denote (1) beliefs in the efficacy of depression treatment and (2) accurate identification of symptoms and their objectively valid appraisal as constituents of an underlying depression.

The reported associations of IB with uptake of depression treatment were adjusted for commonly observed individual-level drivers of help-seeking behaviour such as symptom severity or previous treatment experience.^7^ Independently of these factors, IB explained the uptake of depression treatment. On a descriptive level, our findings suggest that individuals reporting depression-conforming illness identity IB are almost two times as likely to initialise depression treatment during follow-up. This descriptive difference is also evident when adjusting for prevalent DSM-5 diagnosis of depressive disorder. Self-reported previous diagnosis of depression no longer predicted depression treatment uptake when including IB. This points to potential mediation of the effect of previous treatment experiences on depression treatment uptake via IB. As previous research findings suggest people with treatment experience report treatment-congruent causal attributions more frequently (eg, relating to childhood, family and predisposition).^26^

Limited availability of resources or insufficient identification of individuals with depression in primary care are frequently discussed system-level barriers in access to care.^27^ Since we assessed uptake of depression treatment within real-world barriers in access to care, our results stress the role of individual-level predisposing factors for depression treatment uptake. Our findings thus challenge the mechanistic view on health service performance as a function of available resources and efficient distribution.^15^ In line with previous research, our findings suggest that system-level interventions aiming at increasing the availability of healthcare professionals or improving early identification in primary care will be of limited success when failing to address individuals’ agency.^5 6^ Indeed, it appears that individual-level factors such as IB impede or foster uptake of depression treatment, irrespective of results feedback after web-based depression screening or identification and referral in primary care.^4 16^

### IB and depression-related health behaviour

IB indicating a longer duration of symptoms (timeline) predicted less likely engagement in DRHB, such as pursuit of alternative disease management strategies, information seeking or activation of social ties (seeking contact to a medical, or health professional showed a similar, non-significant trend). IB regarding timeline were further related with detrimental symptom changes in all considered domains (quality of life, depression, anxiety and somatic symptom severity). This pattern of findings is in line with previous research linking timeline IB with higher distress and lower functioning in somatic diseases and higher depression severity in newly diagnosed patients.^9 21 28^ Interestingly, while emotional components of IB or IB relating to comprehensibility did not predict depression treatment uptake, more intense concern was associated with more likely engagement in DRHB comprising information seeking. Higher IB regarding comprehensibility and more intense emotions were associated with more likely engagement in activation of social ties. Hence, comprehensibility, concern and emotions may reflect determinants of an individual’s functional level and related agency opposed to seeking and believing in external, professional help.

Previous research suggests variability of IB over time, in response to communication of screening results, with higher personal control and lower behavioural activation after receiving negative compared with positive results of coronary angiography.^29^ Further research has shown that targeted modification of IB is possible in somatic illnesses, for example, targeted text messages not only modified unhelpful IB in younger adults with asthma but also positively affected treatment adherence.^12^ While less is known on targeted IB modification in depression, screening results feedback did not have a significant effect on treatment uptake. Screening in itself may have had an impact on initial IB formation at baseline, as increasing awareness of symptoms belonging to one clinical entity may drive participants to update or initialise their illness-specific IB.^4 30^ Indeed previous findings suggest even subtle online communication may affect cognitive conceptualisations of psychopathology.^31^ Given the existence of web-based screening instruments and given that most people seek information about mental health online, our findings reinforce the clinical relevance of examining modifiability of IB (potentially in relation with screening and feedback) to lower the population-level burden of depression at scale.^19 29^ Our findings further imply that raising awareness and bolstering health literacy regarding depression treatment efficacy may support IB associated with adaptive health behaviours, for example, in case of treatment control beliefs.

### Limitations

Some limitations need to be considered when interpreting reported findings. First, including participants without treatment or diagnosis in the year prior to screening may differentially select a hard-to-reach subpopulation. Thus, power to detect effects of IB regarding uptake of depression treatment may have been reduced and reported findings are likely conservative estimates. Likewise, descriptive characteristics of participants lost-to-follow-up suggest higher treatment control beliefs and more participants with a specific treatment preference or previous history of diagnosis in the analytic sample compared with those lost to follow-up. Second, we cannot differentiate reasons for no uptake of depression treatment (eg, false-positive web-based screening, false-negative testing during help-seeking, no help-seeking, natural remission). Absence of an association of depression severity with uptake of depression treatment may be due to preselection of participants based on reported symptom burden and, thus, related ceiling effects.^20^ Third, given long waiting times for depression care, follow-up time may have been too short to capture uptake of depression treatment. Fourth, despite the study being framed as a survey on mental health, respondents may represent a highly motivated subgroup, which limits generalisability of findings to the general population. Fifth, IB measures vary among studies. While the B-IPQ was cautiously adjusted, we cannot rule out resulting limitations of comparability. Sixth, post hoc power analysis suggests limited power to detect small effects.

## Conclusion

Our findings highlight the importance of IB for the patient journey mounting at the uptake of depression treatment as a final result of help-seeking. We find that IB about consequences, treatment control and illness identity predict uptake of depression treatment in a naturalistic setting.

### Clinical implications

We point to IB as potentially relevant barriers to healthcare utilisation and access to care in currently untreated populations. Future studies may investigate IB as potential intervention target to increase equity in health service utilisation.

## Supplementary material

10.1136/bmjment-2025-301666 online supplemental file 1

## Data Availability

Data are available upon reasonable request.
