# Supplementary material for: Do illness beliefs predict uptake of depression treatment after web-based depression screening? A secondary analysis of the DISCOVER RCT
Source: BMJ Ment Health. 2025 Jul 27;28(1):e301666. doi: 10.1136/bmjment-2025-301666 (PMC12306251; doi:10.1136/bmjment-2025-301666)
Supplement: online supplemental file 1 [file bmjment-28-1-s001.pdf]

Supplementary Material Tables & Figures

Table S1. Illness belief measures in DISCOVER, along with original items from the Brief Illness Perception Questionnaire.

Domain	Brief Illness Perception Questionnaire (Broadbent et al., 2006)	Illness Belief (Kohlmann et al., 2024)	
		Translated (English)	Original (German)
**Consequences[a]**	How much does your illness affect your life?	How much do these complaints affect your life?	Wie stark beeinträchtigen diese Beschwerden Ihr Leben?
**Timeline[a]**	How long do you think your illness will continue?	How long do you think these complaints will continue?	Wie lange meinen Sie, dass diese Beschwerden noch andauern werden?
**Personal Control[a]**	How much control do you feel you have over your illness?	How much control do you feel you have over these complaints?	Wie stark meinen Sie, diese Beschwerden selbst kontrollieren zu können?
**Treatment Control[a]**	How much do you think your treatment can help your illness?	How much do you think a treatment can help with these complaints?	Wie stark meinen Sie, dass eine Behandlung bei diesen Beschwerden helfen kann?
**Illness Identity[a,b]**	How much do you experience symptoms from your illness?	Can you imagine that you are currently suffering from depression?	Können Sie sich vorstellen, aktuell an einer Depression zu leiden?
**Emotional Representation – Concern[a]**	How concerned are you about your illness?	How concerned are you about your complaints?	Wie stark machen Sie sich Sorgen über diese Beschwerden?
**Illness Comprehensibility[a]**	How well do you feel you understand your illness?	How well do you feel you understand these complaints?	Wie gut meinen Sie, diese Beschwerden zu verstehen?
**Emotional Representation – Emotions[a]**	How much does your illness affect you emotionally? (e.g. does it make you angry, scared, upset or depressed?)	How much do these complaints affect you emotionally? (e.g., do they make you angry, scared, upset or depressed?) (emotional representation – emotions)	Wie stark sind Sie durch diese Beschwerden gefühlsmäßig beeinträchtigt? Sind Sie durch Ihre Beschwerden zum Beispiel ärgerlich, verängstigt, aufgewühlt oder niedergeschlagen?
**Temporality[b]**	n/a	Is this the first time in your life that you are experiencing such complaints?	Ist das das erste Mal in Ihrem Leben, dass Sie solche Beschwerden erleben?

[a]Items are rated from 0 to 10 with 10 reflecting highest agreement, both in original version of B-IPQ and in the adapted version used in this study.
[b]Binary item in the adapted version of the B-IPQ used in this study.

Table S2. Odds ratios for uptake of depression treatment at six months' follow-up without illness beliefs.

Term	OR	SE	P-value	Lower CI	Upper CI
**Condition**					
No Feedback	Reference				
Non-tailored Feedback	1.28	0.20	.22	0.86	1.89
Tailored Feedback	1.38	0.20	.10	0.94	2.03
**Depression severity (PHQ-9)**	1.03	0.02	.15	0.99	1.08
**Treatment Preference**					
Unspecified	Reference				
General Practitioner or Psychotherapist	2.46	0.22	<.001	1.61	3.86
**Previous Lifetime Diagnosis of Depression/Burnout**					
No Previous Diagnosis	Reference				
Previous Diagnosis	1.45	0.17	.03	1.04	2.02
**Formal Education**					
Low (<10 years)	Reference				
Middle (≥10 years)	1.14	0.26	.62	0.69	1.89
High (university entrance qualification)	1.42	0.26	.17	0.86	2.38
**Gender**					
Female	Reference				
Male	0.97	0.19	.86	0.66	1.40
**Age**	1.02	0.01	.02	1.00	1.03
**Living Status**					
With Others	Reference				
Alone	1.00	0.17	.99	0.71	1.40
**Working Status**					
Not Working	Reference				
Retired	1.20	0.40	.66	0.54	2.65
Unemployed	2.52	0.40	.02	1.14	5.60
Shift Work/Hourly Rate	0.91	0.29	.76	0.52	1.64
Employed	1.28	0.27	.37	0.76	2.23
**SCID Screening**					
Negative	Reference				
Positive	1.64	0.18	.007	1.14	2.35

Note. OR = Odds Ratio, PHQ-9 = Patient Health Questionnaire-9, GP = General Practitioner, PT = Psychotherapist, SCID = Structured Clinical Interview for DSM Disorders.

Table S3. Descriptive characteristics in the primary analysis set and those lost-to-follow-up.

Characteristic	Primary Analysis Set (N=871)	Lost-To-Follow-Up (N=137)	P-value
**Age, Mean (SD)**	37.5 (14.0)	38.8 (15.1)	.34
**Gender**			.008
Male, No. (%)	237 (27.2%)	53 (38.7%)	
Female, No. (%)	634 (72.8%)	84 (61.3%)	
**Formal Education**			<.001
Low (<10 years), No. (%)	144 (16.5%)	41 (29.9%)	
Middle (≥10 years), No. (%)	262 (30.1%)	56 (40.9%)	
High (university entrance qualification), No. (%)	465 (53.4%)	40 (29.2%)	
**Working Status**			.03
Not Working, No. (%)	116 (13.3%)	19 (13.9%)	
Retired, No. (%)	69 (7.9%)	21 (15.3%)	
Unemployed, No. (%)	44 (5.1%)	9 (6.6%)	
Shift Work/Hourly Rate, No. (%)	249 (28.6%)	29 (21.2%)	
Employed	393 (45.1%)	58 (42.3%)	
Missing[a]		1 (0.7%)	
**Living Status**			.81
With others, No. (%)	585 (67.2%)	90 (65.7%)	
Alone, No. (%)	286 (32.8%)	47 (34.3%)	
**Treatment Preference**			.003
Unspecified, No. (%)	200 (23.0%)	48 (35.0%)	
General Practitioner or Psychotherapist, No. (%)	671 (77.0%)	89 (65.0%)	
**Previous Lifetime Diagnosis of Depression/Burnout**			.18
No Previous Diagnosis, No. (%)	510 (58.6%)	89 (65.0%)	
Previous Diagnosis, No. (%)	361 (41.4%)	48 (35.0%)	
**Depression severity (PHQ-9), Mean (SD)**	14.7 (3.91)	15.0 (4.51)	.54
**SCID Interview**			.99
Negative, No. (%)	327 (37.5%)	1 (0.7%)	
Positive, No. (%)	544 (62.5%)	1 (0.7%)	
Missing[a]		135 (98.5%)	
**Condition**			.48
No Feedback, No. (%)	298 (34.2%)	43 (31.4%)	
Non-tailored Feedback, No. (%)	285 (32.7%)	52 (38.0%)	
Tailored Feedback, No. (%)	288 (33.1%)	42 (30.7%)	
**Illness Beliefs**			
Consequences[b], Mean (SD)	6.39 (2.05)	6.34 (2.21)	.77
Timeline[b], Mean (SD)	6.63 (2.27)	7.18 (2.34)	.01
Personal Control[b], Mean (SD)	4.89 (2.19)	4.61 (2.34)	.18
Treatment Control[b], Mean (SD)	6.21 (2.67)	5.45 (2.43)	<.001
Concern[b], Mean (SD)	6.51 (2.37)	6.50 (2.30)	.98
Comprehensibility[b], Mean (SD)	6.57 (2.34)	6.09 (2.30)	.03
Emotions[b], Mean (SD)	7.38 (1.99)	7.16 (2.04)	.23
Temporality (previous experience vs. first time)[c], No. (%)	687 (78.9%)	114 (83.2%)	.22
Illness Identity (yes vs. no/maybe)[d], No. (%)	360 (41.3%)	52 (38.0%)	.51

Note. PHQ-9 = Patient Health Questionnaire-9, SCID = Structured Clinical Interview for DSM Disorders.
[a]Number of missings reported for transparency. These are not included in the statistical comparison.
[b]Items are rated from 0 to 10 with 10 reflecting highest agreement with statements listed in Table S1.
[c]Binary item indicating if participants experienced the complaints before. For one person, temporality was missing. Not included in the table due to spacing.
[d]Binary item indicating if participants appraise their symptoms to probable depression.

Table S4. Odds ratios for uptake of depression treatment at six months' follow-up without adjustment for SCID.

Term	OR	SE	P-value	Lower CI	Upper CI
**Illness Belief**					
Consequences[a]	1.09	0.05	.12	0.98	1.21
Timeline[a]	0.98	0.04	.56	0.90	1.06
Personal Control[a]	0.92	0.04	.03	0.86	0.99
Treatment Control[a]	1.19	0.04	<.001	1.11	1.27
Concern[a]	0.99	0.05	.90	0.91	1.09
Comprehensibility[a]	0.98	0.04	.52	0.91	1.05
Emotions[a]	1.04	0.05	.48	0.94	1.15
Temporality (previous experience)[b]	1.04	0.21	.84	0.70	1.57
Illness Identity (yes)[c]	1.60	0.17	.007	1.14	2.25
**Condition**					
No Feedback	Reference				
Non-tailored Feedback	1.27	0.19	.21	0.87	1.85
Tailored Feedback	1.30	0.19	.17	0.90	1.88
**Depression severity (PHQ-9)**	0.99	0.02	.59	0.94	1.03
**Treatment Preference**					
Unspecified	Reference				
General Practitioner or Psychotherapist	1.95	0.21	.001	1.31	2.98
**Previous Lifetime Diagnosis of Depression/Burnout**					
No Previous Diagnosis	Reference				
Previous Diagnosis	1.44	0.17	.03	1.03	2.02
**Formal Education**					
Low (<10 years)	Reference				
Middle (≥10 years)	1.13	0.24	.61	0.71	1.83
High (university entrance qualification)	1.27	0.25	.34	0.78	2.10
**Gender**					
Female	Reference				
Male	1.02	0.18	.93	0.71	1.46
**Age**	1.01	0.01	.09	1.00	1.03
**Living Status**					
With Others	Reference				
Alone	1.06	0.17	.71	0.77	1.47
**Working Status**					
Not Working	Reference				
Retired	2.17	0.38	.04	1.03	4.64
Unemployed	2.49	0.39	.02	1.16	5.39
Shift Work/Hourly Rate	1.10	0.29	.74	0.63	1.95
Employed	1.40	0.27	.21	0.83	2.42

Note. OR = Odds Ratio, PHQ-9 = Patient Health Questionnaire-9, GP = General Practitioner, PT = Psychotherapist, SCID = Structured Clinical Interview for DSM Disorders.

[a]Items are rated from 0 to 10 with 10 reflecting highest agreement with statements listed in Table S1.

[b]Binary item indicating if participants experienced the complaints before. With the reference level *first time*, OR indicates greater uptake in those with previous experience.

[c]Binary item indicating if participants appraise their symptoms to probable depression. With the reference level *no/maybe*, OR indicates greater uptake in those with a depression-conforming illness identity belief.

Table S5. Post hoc power analysis parameters and achieved power for significant illness beliefs.

Parameter	Treatment Control	Consequences	Illness Identity
**Tail(s)**	Two	Two	Two
**Odds Ratio**	1.19	1.12	1.65
**Pr(Y=1 | X=1), H_0[a]**	0.27	0.27	0.27
**α Error Probability**	0.05	0.05	0.05
**Total Sample Size**	871	871	871
**R^2 (Other Predictors)**	0.22	0.48	0.21
**X Distribution**	Normal	Normal	Binomial
**X Parameters**	$\mu = 6.21$, $\sigma = 2.67$	$\mu = 6.39$, $\sigma = 2.05$	$\pi = 0.41$
**Critical z**	1.96	1.96	1.96
**Power (1−β)**	0.99	0.67	0.85

Note. Post hoc power analyses were conducted using G*Power 3.1.9.7, employing the large sample z-test as recommended by Demidenko (2007) with variance correction. Values are rounded for clarity.
[a]Denotes the mean outcome probability assuming the null hypothesis of no effect of the predictor.

Table S6. Odds ratios for depression-related health behaviour – help at six months' follow-up.

Term	OR	SE	P-value	Lower CI	Upper CI
**Illness Belief**					
Consequences[a]	1.07	0.05	.16	0.97	1.19
Timeline[a]	0.95	0.04	.19	0.88	1.03
Personal Control[a]	0.90	0.04	.006	0.84	0.97
Treatment Control[a]	1.17	0.03	<.001	1.10	1.25
Concern[a]	1.00	0.04	.91	0.92	1.09
Comprehensibility[a]	0.97	0.03	.40	0.91	1.04
Emotions[a]	1.05	0.05	.34	0.95	1.16
Temporality (previous experience)[b]	1.03	0.19	.89	0.71	1.50
Illness Identity (yes)[c]	1.42	0.17	.04	1.03	1.98
**Condition**					
No Feedback	Reference				
Non-tailored Feedback	1.17	0.18	.39	0.82	1.68
Tailored Feedback	1.35	0.18	.10	0.95	1.93
**Depression severity (PHQ-9)**	1.00	0.02	.88	0.95	1.05
**Treatment Preference**					
Unspecified	Reference				
General Practitioner or Psychotherapist	1.90	0.19	<.001	1.32	2.77
**Previous Lifetime Diagnosis of Depression/Burnout**					
No Previous Diagnosis	Reference				
Previous Diagnosis	1.22	0.17	.23	0.88	1.70
**Formal Education**					
Low (<10 years)	Reference				
Middle (≥10 years)	1.30	0.24	.28	0.81	2.09
High (university entrance qualification)	0.93	0.24	.77	0.58	1.51
**Gender**					
Female	Reference				
Male	0.98	0.18	.91	0.69	1.39
**Age**	1.02	0.01	.02	1.00	1.03
**Living Status**					
With Others	Reference				
Alone	0.88	0.16	.41	0.63	1.20
**Working Status**					
Not Working	Reference				
Retired	1.13	0.38	.75	0.54	2.39
Unemployed	0.94	0.39	.88	0.44	2.05
Shift Work/Hourly Rate	0.69	0.25	.14	0.42	1.13
Employed	0.78	0.24	.29	0.48	1.25
**SCID Screening**					
Negative	Reference				
Positive	1.42	0.16	.03	1.03	1.96

Note. OR = Odds Ratio, PHQ-9 = Patient Health Questionnaire-9, GP = General Practitioner, PT = Psychotherapist, SCID = Structured Clinical Interview for DSM Disorders.

[a]Items are rated from 0 to 10 with 10 reflecting highest agreement with statements listed in Table S1.

[b]Binary item indicating if participants experienced the complaints before. With the reference level *first time*, OR indicates greater uptake in those with previous experience.

[c]Binary item indicating if participants appraise their symptoms to probable depression. With the reference level *no/maybe*, OR indicates greater uptake in those with a depression-conforming illness identity belief.

Table S7. Odds ratios for depression-related health behaviour – manage at six months' follow-up.

Term	OR	SE	P-value	Lower CI	Upper CI
**Illness Belief**					
Consequences[a]	1.04	0.05	.45	0.94	1.15
Timeline[a]	0.88	0.04	.002	0.81	0.95
Personal Control[a]	1.01	0.04	.80	0.94	1.08
Treatment Control[a]	1.07	0.03	.03	1.01	1.14
Concern[a]	1.07	0.04	.11	0.98	1.17
Comprehensibility[a]	1.02	0.03	.63	0.95	1.09
Emotions[a]	1.05	0.05	.31	0.95	1.16
Temporality (previous experience)[b]	0.95	0.20	.80	0.64	1.39
Illness Identity (yes)[c]	0.80	0.17	.17	0.56	1.11
**Condition**					
No Feedback	Reference				
Non-tailored Feedback	1.12	0.19	.53	0.78	1.62
Tailored Feedback	1.36	0.19	.09	0.95	1.96
**Depression severity (PHQ-9)**	0.97	0.02	.28	0.93	1.02
**Treatment Preference**					
Unspecified	Reference				
General Practitioner or Psychotherapist	1.26	0.18	.20	0.88	1.80
**Previous Lifetime Diagnosis of Depression/Burnout**					
No Previous Diagnosis	Reference				
Previous Diagnosis	1.42	0.17	.04	1.01	2.01
**Formal Education**					
Low (<10 years)	Reference				
Middle (≥10 years)	1.87	0.24	.008	1.18	2.98
High (university entrance qualification)	2.57	0.24	<.001	1.60	4.13
**Gender**					
Female	Reference				
Male	0.57	0.18	.001	0.40	0.80
**Age**	0.99	0.01	.44	0.98	1.01
**Living Status**					
With Others	Reference				
Alone	1.17	0.17	.34	0.85	1.63
**Working Status**					
Not Working	Reference				
Retired	0.84	0.38	.65	0.40	1.78
Unemployed	1.33	0.41	.48	0.61	3.00
Shift Work/Hourly Rate	0.96	0.25	.87	0.58	1.57
Employed	1.10	0.24	.70	0.68	1.77
**SCID Screening**					
Negative	Reference				
Positive	1.02	0.17	.89	0.73	1.42

Note. OR = Odds Ratio, PHQ-9 = Patient Health Questionnaire-9, GP = General Practitioner, PT = Psychotherapist, SCID = Structured Clinical Interview for DSM Disorders.

[a]Items are rated from 0 to 10 with 10 reflecting highest agreement with statements listed in Table S1.

[b]Binary item indicating if participants experienced the complaints before. With the reference level *first time*, OR indicates greater uptake in those with previous experience.

[c]Binary item indicating if participants appraise their symptoms to probable depression. With the reference level *no/maybe*, OR indicates greater uptake in those with a depression-conforming illness identity belief.

Table S8. Odds ratios for depression-related health behaviour – info at six months' follow-up.

Term	OR	SE	P-value	Lower CI	Upper CI
**Illness Belief**					
Consequences[a]	1.02	0.05	.70	0.92	1.13
Timeline[a]	0.92	0.04	.045	0.85	1.00
Personal Control[a]	0.99	0.04	.87	0.93	1.07
Treatment Control[a]	1.05	0.03	.14	0.98	1.12
Concern[a]	1.11	0.04	.01	1.02	1.21
Comprehensibility[a]	0.97	0.03	.38	0.91	1.04
Emotions[a]	0.98	0.05	.76	0.89	1.09
Temporality (previous experience)[b]	0.91	0.19	.62	0.62	1.32
Illness Identity (yes)[c]	1.61	0.17	.005	1.16	2.25
**Condition**					
No Feedback	Reference				
Non-tailored Feedback	0.94	0.18	.74	0.66	1.35
Tailored Feedback	0.98	0.18	.92	0.69	1.40
**Depression severity (PHQ-9)**	1.04	0.02	.14	0.99	1.09
**Treatment Preference**					
Unspecified	Reference				
General Practitioner or Psychotherapist	0.88	0.18	.50	0.61	1.27
**Previous Lifetime Diagnosis of Depression/Burnout**					
No Previous Diagnosis	Reference				
Previous Diagnosis	1.09	0.17	.61	0.778	1.52
**Formal Education**					
Low (<10 years)	Reference				
Middle (≥10 years)	1.78	0.25	.02	1.10	2.89
High (university entrance qualification)	1.53	0.25	.08	0.95	2.49
**Gender**					
Female	Reference				
Male	0.72	0.18	.07	0.51	1.03
**Age**	0.99	0.01	.045	0.97	1.00
**Living Status**					
With Others	Reference				
Alone	0.97	0.16	.84	0.71	1.33
**Working Status**					
Not Working	Reference				
Retired	0.84	0.39	.65	0.39	1.80
Unemployed	0.96	0.40	.92	0.44	2.10
Shift Work/Hourly Rate	1.11	0.25	.67	0.68	1.80
Employed	1.26	0.24	.33	0.79	2.02
**SCID Screening**					
Negative	Reference				
Positive	1.79	0.16	<.001	1.30	2.46

Note. OR = Odds Ratio, PHQ-9 = Patient Health Questionnaire-9, GP = General Practitioner, PT = Psychotherapist, SCID = Structured Clinical Interview for DSM Disorders.

[a]Items are rated from 0 to 10 with 10 reflecting highest agreement with statements listed in Table S1.

[b]Binary item indicating if participants experienced the complaints before. With the reference level *first time*, OR indicates greater uptake in those with previous experience.

[c]Binary item indicating if participants appraise their symptoms to probable depression. With the reference level *no/maybe*, OR indicates greater uptake in those with a depression-conforming illness identity belief.

Table S9. Odds ratios for depression-related health behaviour – social at six months' follow-up.

Term	OR	SE	P-value	Lower CI	Upper CI
**Illness Belief**					
Consequences[a]	0.93	0.05	.15	0.83	1.03
Timeline[a]	0.91	0.04	.03	0.84	0.99
Personal Control[a]	0.98	0.04	.67	0.91	1.06
Treatment Control[a]	1.07	0.03	.045	1.00	1.14
Concern[a]	1.02	0.04	.59	0.94	1.12
Comprehensibility[a]	1.12	0.04	.001	1.05	1.20
Emotions[a]	1.14	0.05	.01	1.03	1.26
Temporality (previous experience)[b]	1.10	0.20	.62	0.74	1.63
Illness Identity (yes)[c]	1.38	0.18	.08	0.96	1.97
**Condition**					
No Feedback	Reference				
Non-tailored Feedback	1.12	0.19	.56	0.77	1.63
Tailored Feedback	1.29	0.19	.19	0.88	1.88
**Depression severity (PHQ-9)**	0.94	0.03	.02	0.90	0.99
**Treatment Preference**					
Unspecified	Reference				
General Practitioner or Psychotherapist	1.45	0.19	.047	1.00	2.08
**Previous Lifetime Diagnosis of Depression/Burnout**					
No Previous Diagnosis	Reference				
Previous Diagnosis	1.42	0.18	.050	1.00	2.03
**Formal Education**					
Low (<10 years)	Reference				
Middle (≥10 years)	1.80	0.24	.02	1.12	2.89
High (university entrance qualification)	2.39	0.25	<.001	1.48	3.88
**Gender**					
Female	Reference				
Male	0.67	0.18	.03	0.47	0.95
**Age**	0.98	0.01	.002	0.96	0.99
**Living Status**					
With Others	Reference				
Alone	1.23	0.17	.22	0.88	1.73
**Working Status**					
Not Working	Reference				
Retired	1.21	0.39	.62	0.56	2.62
Unemployed	0.88	0.42	.77	0.39	2.04
Shift Work/Hourly Rate	0.76	0.27	.31	0.44	1.29
Employed	0.75	0.26	.27	0.44	1.24
**SCID Screening**					
Negative	Reference				
Positive	1.11	0.17	.54	0.79	1.57

Note. OR = Odds Ratio, PHQ-9 = Patient Health Questionnaire-9, GP = General Practitioner, PT = Psychotherapist, SCID = Structured Clinical Interview for DSM Disorders.

[a]Items are rated from 0 to 10 with 10 reflecting highest agreement with statements listed in Table S1.

[b]Binary item indicating if participants experienced the complaints before. With the reference level *first time*, OR indicates greater uptake in those with previous experience.

[c]Binary item indicating if participants appraise their symptoms to probable depression. With the reference level *no/maybe*, OR indicates greater uptake in those with a depression-conforming illness identity belief.

Table S10. Odds ratios for diagnoses of depression or burnout at six months' follow-up.

Term	OR	SE	P-value	Lower CI	Upper CI
**Illness Belief**					
Consequences[a]	1.23	0.08	.007	1.06	1.42
Timeline[a]	0.91	0.06	.12	0.81	1.02
Personal Control[a]	0.86	0.05	.005	0.78	0.96
Treatment Control[a]	1.26	0.05	<.001	1.15	1.40
Concern[a]	0.96	0.06	.46	0.85	1.08
Comprehensibility[a]	0.98	0.05	.76	0.89	1.09
Emotions[a]	0.96	0.07	.59	0.83	1.11
Temporality (previous experience)[b]	1.24	0.29	.45	0.72	2.21
Illness Identity (yes)[c]	2.88	0.24	<.001	1.82	4.62
**Condition**					
No Feedback	Reference				
Non-tailored Feedback	1.34	0.27	.27	0.80	2.26
Tailored Feedback	1.37	0.26	.22	0.83	2.27
**Depression severity (PHQ-9)**	1.02	0.03	.58	0.95	1.09
**Treatment Preference**					
Unspecified	Reference				
General Practitioner or Psychotherapist	1.84	0.30	.04	1.04	3.40
**Previous Lifetime Diagnosis of Depression/Burnout**					
No Previous Diagnosis	Reference				
Previous Diagnosis	1.50	0.24	.09	0.94	2.40
**Formal Education**					
Low (<10 years)	Reference				
Middle (≥10 years)	1.38	0.35	.35	0.70	2.77
High (university entrance qualification)	1.37	0.35	.36	0.70	2.76
**Gender**					
Female	Reference				
Male	1.19	0.26	.49	0.72	1.96
**Age**	1.01	0.01	.13	1.00	1.03
**Living Status**					
With Others	Reference				
Alone	1.33	0.23	.20	0.85	2.07
**Working Status**					
Not Working	Reference				
Retired	0.79	0.55	.66	0.26	2.30
Unemployed	1.76	0.51	.27	0.64	4.77
Shift Work/Hourly Rate	0.96	0.37	.90	0.47	2.01
Employed	0.83	0.35	.59	0.42	1.69
**SCID Screening**					
Negative	Reference				
Positive	1.31	0.25	.28	0.81	2.15

Note. OR = Odds Ratio, PHQ-9 = Patient Health Questionnaire-9, GP = General Practitioner, PT = Psychotherapist, SCID = Structured Clinical Interview for DSM Disorders.

[a]Items are rated from 0 to 10 with 10 reflecting highest agreement with statements listed in Table S1.

[b]Binary item indicating if participants experienced the complaints before. With the reference level *first time*, OR indicates greater uptake in those with previous experience.

[c]Binary item indicating if participants appraise their symptoms to probable depression. With the reference level *no/maybe*, OR indicates greater uptake in those with a depression-conforming illness identity belief.

Table S11. Regression coefficient for health-related quality of life change at six months' follow-up.

Term	RC	SE	P-value	Lower CI	Upper CI
**Illness Belief**					
Consequences[a]	-0.95	0.46	.04	-1.86	-0.05
Timeline[a]	-1.39	0.36	<.001	-2.10	-0.68
Personal Control[a]	0.41	0.32	.21	-0.23	1.04
Treatment Control[a]	0.13	0.28	.65	-0.42	0.68
Concern[a]	0.09	0.38	.82	-0.66	0.83
Comprehensibility[a]	0.12	0.30	.69	-0.47	0.71
Emotions[a]	-0.51	0.45	.25	-1.37	0.35
Temporality (previous experience)[b]	0.13	1.71	.94	-3.22	3.48
Illness Identity (yes)[c]	-1.93	1.52	.21	-4.91	1.05
**Condition**					
No Feedback	Reference				
Non-tailored Feedback	-2.13	1.64	.20	-5.35	1.09
Tailored Feedback	-0.88	1.61	.59	-4.03	2.27
**Depression severity (PHQ-9)**	-0.57	0.22	.01	-1.00	-0.13
**Treatment Preference**					
Unspecified	Reference				
General Practitioner or Psychotherapist	-2.89	1.63	.08	-6.09	0.30
**Previous Lifetime Diagnosis of Depression/Burnout**					
No Previous Diagnosis	Reference				
Previous Diagnosis	-2.42	1.51	.11	-5.37	0.53
**Formal Education**					
Low (<10 years)	Reference				
Middle (≥10 years)	-0.61	2.19	.78	-4.91	3.68
High (university entrance qualification)	-0.43	2.22	.85	-4.76	3.92
**Gender**					
Female	Reference				
Male	-3.41	1.61	.04	-6.56	-0.25
**Age**	-0.04	0.06	.49	-0.16	0.08
**Living Status**					
With Others	Reference				
Alone	-1.35	1.43	.35	-4.16	1.46
**Working Status**					
Not Working	Reference				
Retired	-2.11	3.43	.54	-8.84	4.62
Unemployed	10.09	3.60	.005	3.03	17.14
Shift Work/Hourly Rate	4.17	2.22	.06	-0.19	8.53
Employed	7.52	2.13	<.001	3.34	11.70
**SCID Screening**					
Negative	Reference				
Positive	-2.06	1.47	.16	-4.95	0.83
**Health-Related Quality of Life (EQ-5D-5L)**	-0.81	0.03	<.001	-0.88	-0.75

Note. RC = Regression Coefficient, PHQ-9 = Patient Health Questionnaire-9, GP = general practitioner, PT = Psychotherapist, SCID = Structured Clinical Interview for DSM Disorders, EQ-5D-5L = health-related quality of life assessed with the EQ-5D-5L visual analogue scale.

[a]Items are rated from 0 to 10 with 10 reflecting highest agreement with statements listed in Table S1.

[b]Binary item indicating if participants experienced the complaints before. With the reference level *first time*, OR indicates greater uptake in those with previous experience.

[c]Binary item indicating if participants appraise their symptoms to probable depression. With the reference level *no/maybe*, OR indicates greater uptake in those with a depression-conforming illness identity belief.

Table S12. Regression coefficient for depression severity change at six months' follow-up.

Term	RC	SE	P-value	Lower CI	Upper CI
**Illness Belief**					
Consequences[a]	0.12	0.11	.26	-0.09	0.34
Timeline[a]	0.27	0.09	.002	0.10	0.45
Personal Control[a]	0.02	0.08	.80	-0.13	0.17
Treatment Control[a]	-0.01	0.07	.85	-0.15	0.12
Concern[a]	0.05	0.09	.55	-0.13	0.24
Comprehensibility[a]	0.03	0.07	.69	-0.11	0.17
Emotions[a]	0.04	0.11	.72	-0.17	0.25
Temporality (previous experience)[b]	0.34	0.42	.42	-0.48	1.15
Illness Identity (yes)[c]	0.41	0.37	.27	-0.31	1.14
**Condition**					
No Feedback	Reference				
Non-tailored Feedback	0.05	0.40	.90	-0.73	0.83
Tailored Feedback	-0.14	0.39	.71	-0.91	0.63
**Depression severity (PHQ-9)**	-0.56	0.05	<.001	-0.66	-0.45
**Treatment Preference**					
Unspecified	Reference				
General Practitioner or Psychotherapist	0.24	0.40	.55	-0.54	1.02
**Previous Lifetime Diagnosis of Depression/Burnout**					
No Previous Diagnosis	Reference				
Previous Diagnosis	0.95	0.37	.01	0.23	1.67
**Formal Education**					
Low (<10 years)	Reference				
Middle (≥10 years)	-0.52	0.53	.33	-1.56	0.52
High (university entrance qualification)	-1.14	0.53	.03	-2.18	-0.09
**Gender**					
Female	Reference				
Male	-0.20	0.39	.61	-0.97	0.56
**Age**	-0.02	0.02	.17	-0.05	0.01
**Living Status**					
With Others	Reference				
Alone	0.23	0.35	.51	-0.46	0.92
**Working Status**					
Not Working	Reference				
Retired	-0.74	0.84	.38	-2.38	0.91
Unemployed	-1.88	0.87	.03	-3.58	-0.18
Shift Work/Hourly Rate	-1.02	0.54	.06	-2.08	0.04
Employed	-1.55	0.52	.003	-2.57	-0.53
**SCID Screening**					
Negative	Reference				
Positive	1.51	0.36	<.001***	0.81	2.22

Note. RC = Regression Coefficient, PHQ-9 = Patient Health Questionnaire-9, GP = general practitioner, PT = Psychotherapist, SCID = Structured Clinical Interview for DSM Disorders.

[a]Items are rated from 0 to 10 with 10 reflecting highest agreement with statements listed in Table S1.

[b]Binary item indicating if participants experienced the complaints before. With the reference level *first time*, OR indicates greater uptake in those with previous experience.

[c]Binary item indicating if participants appraise their symptoms to probable depression. With the reference level *no/maybe*, OR indicates greater uptake in those with a depression-conforming illness identity belief.

Table S13. Regression coefficient for anxiety severity change at six months' follow-up.

Term	RC	SE	P-value	Lower CI	Upper CI
**Illness Belief**					
Consequences[a]	0.03	0.10	.77	-0.17	0.22
Timeline[a]	0.18	0.08	.02	0.03	0.34
Personal Control[a]	-0.01	0.07	.85	-0.15	0.12
Treatment Control[a]	-0.02	0.06	.77	-0.14	0.10
Concern[a]	0.08	0.08	.34	-0.08	0.24
Comprehensibility[a]	0.06	0.06	.36	-0.07	0.19
Emotions[a]	0.12	0.10	.21	-0.07	0.31
Temporality (previous experience)[b]	0.50	0.37	.18	-0.22	1.22
Illness Identity (yes)[c]	0.14	0.33	.68	-0.51	0.78
**Condition**					
No Feedback	Reference				
Non-tailored Feedback	0.48	0.35	.17	-0.21	1.18
Tailored Feedback	-0.00	0.35	.99	-0.68	0.68
**Depression severity (PHQ-9)**	0.15	0.05	.002	0.05	0.25
**Treatment Preference**					
Unspecified	Reference				
General Practitioner or Psychotherapist	0.12	0.35	.74	-0.58	0.81
**Previous Lifetime Diagnosis of Depression/Burnout**					
No Previous Diagnosis	Reference				
Previous Diagnosis	0.40	0.33	.21	-0.23	1.04
**Formal Education**					
Low (<10 years)	Reference				
Middle (≥10 years)	0.08	0.47	.86	-0.85	1.01
High (university entrance qualification)	-0.51	0.48	.29	-1.44	0.43
**Gender**					
Female	Reference				
Male	0.02	0.35	.97	-0.67	0.70
**Age**	-0.03	0.01	.01	-0.05	-0.00
**Living Status**					
With Others	Reference				
Alone	-0.13	0.31	.68	-0.74	0.48
**Working Status**					
Not Working	Reference				
Retired	0.59	0.74	.43	-0.87	2.05
Unemployed	-2.25	0.78	.004	-3.78	-0.73
Shift Work/Hourly Rate	-0.52	0.48	.27	-1.46	0.41
Employed	-0.76	0.46	.10	-1.66	0.14
**SCID Screening**					
Negative	Reference				
Positive	0.93	0.32	.003	0.31	1.56
**Anxiety Severity (GAD-7)**	-0.69	0.04	<.001	-0.77	-0.61

Note. RC = Regression Coefficient, PHQ-9 = Patient Health Questionnaire-9, GP = general practitioner, PT = Psychotherapist, SCID = Structured Clinical Interview for DSM Disorders, GAD-7 = Generalized Anxiety Disorder Scale-7.

[a]Items are rated from 0 to 10 with 10 reflecting highest agreement with statements listed in Table S1.

[b]Binary item indicating if participants experienced the complaints before. With the reference level *first time*, OR indicates greater uptake in those with previous experience.

[c]Binary item indicating if participants appraise their symptoms to probable depression. With the reference level *no/maybe*, OR indicates greater uptake in those with a depression-conforming illness identity belief.

Table S14. Regression coefficient for somatic symptom severity change at six months' follow-up.

Term	RC	SE	P-value	Lower CI	Upper CI
**Illness Belief**					
Consequences[a]	0.03	0.12	.82	-0.20	0.25
Timeline[a]	0.37	0.09	<.001	0.20	0.55
Personal Control[a]	0.07	0.08	.41	-0.09	0.22
Treatment Control[a]	0.06	0.07	.38	-0.08	0.20
Concern[a]	-0.02	0.09	.85	-0.20	0.17
Comprehensibility[a]	0.00	0.07	.96	-0.14	0.15
Emotions[a]	-0.02	0.11	.84	-0.24	0.19
Temporality (previous experience)[b]	0.39	0.43	.37	-0.45	1.22
Illness Identity (yes)[c]	-0.01	0.38	.97	-0.76	0.73
**Condition**					
No Feedback	Reference				
Non-tailored Feedback	0.72	0.41	.08	-0.08	1.52
Tailored Feedback	0.15	0.40	.70	-0.63	0.94
**Depression severity (PHQ-9)**	0.12	0.06	.04	0.01	0.23
**Treatment Preference**					
Unspecified	Reference				
General Practitioner or Psychotherapist	0.15	0.41	.71	-0.65	0.95
**Previous Lifetime Diagnosis of Depression/Burnout**					
No Previous Diagnosis	Reference				
Previous Diagnosis	0.89	0.38	.02	0.16	1.63
**Formal Education**					
Low (<10 years)	Reference				
Middle (≥10 years)	-0.89	0.55	.10	-1.97	0.18
High (university entrance qualification)	-1.53	0.55	.006	-2.62	-0.44
**Gender**					
Female	Reference				
Male	-0.65	0.40	.11	-1.44	0.14
**Age**	-0.03	0.02	.04	-0.06	-0.00
**Living Status**					
With Others	Reference				
Alone	-0.56	0.36	.12	-1.26	0.14
**Working Status**					
Not Working	Reference				
Retired	1.50	0.86	.08	-0.19	3.19
Unemployed	-1.29	0.90	.15	-3.05	0.48
Shift Work/Hourly Rate	-0.04	0.55	.94	-1.13	1.04
Employed	-0.37	0.53	.48	-1.42	0.67
**SCID Screening**					
Negative	Reference				
Positive	0.74	0.37	.045	0.02	1.46
**Somatic Symptom Severity (SSS-8)**	-0.48	0.04	<.001	-0.55	-0.40

Note. RC = Regression Coefficient, PHQ-9 = Patient Health Questionnaire-9, GP = general practitioner, PT = Psychotherapist, SCID = Structured Clinical Interview for DSM Disorders, SSS-8 = Somatic Symptom Scale-8.
[a]Items are rated from 0 to 10 with 10 reflecting highest agreement with statements listed in Table S1.
[b]Binary item indicating if participants experienced the complaints before. With the reference level *first time*, OR indicates greater uptake in those with previous experience.
[c]Binary item indicating if participants appraise their symptoms to probable depression. With the reference level *no/maybe*, OR indicates greater uptake in those with a depression-conforming illness identity belief.

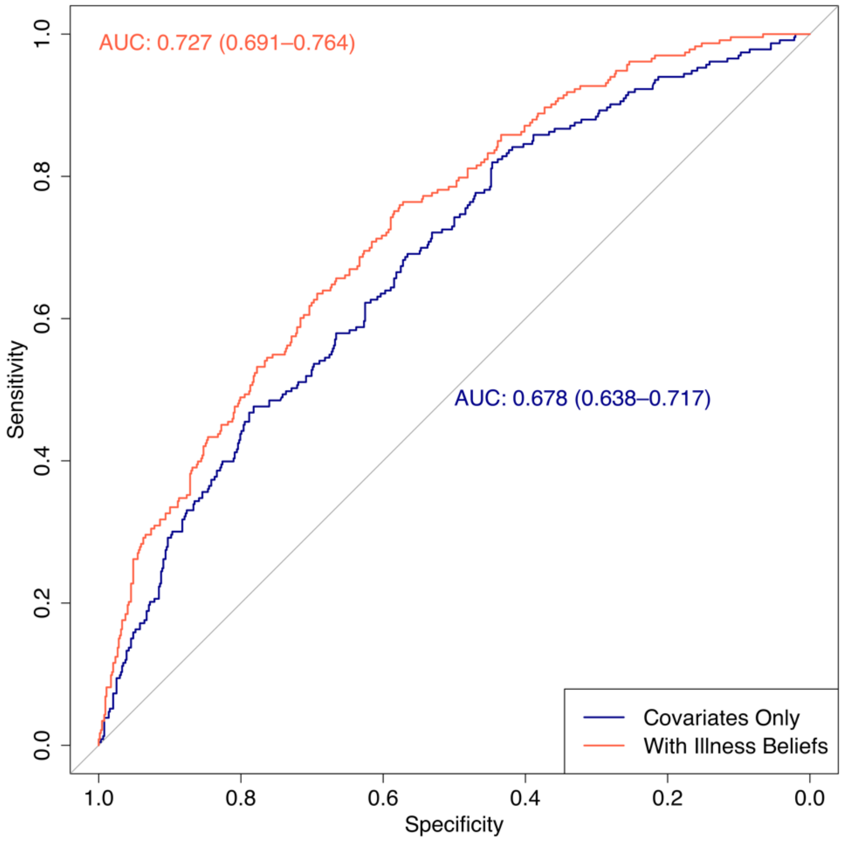

Figure S1. Area under the receiver-operating characteristic curve (AUC) with 95% confidence intervals for the logistic regression of uptake of depression treatment in the primary analysis model with illness beliefs (orange) and the covariate only model (navy).

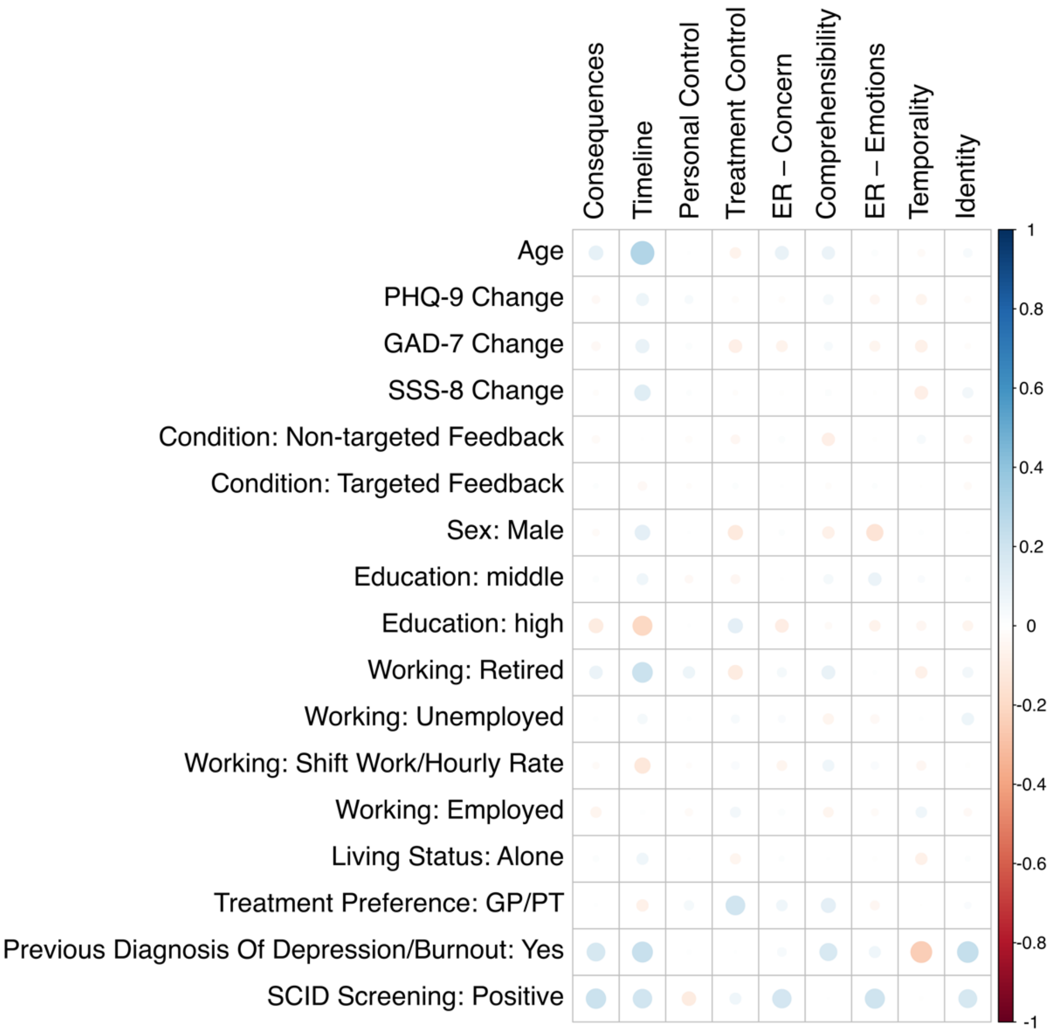

Figure S2. Bivariate association of dichotomized covariates with illness beliefs. Correlation varies from 1 (blue) to -1 (red).
